# Supplementary material for: Multimodal anti-Helicobacter pylori effects of Lactobacillus casei HY001: Evidence from in vitro and in vivo studies
Source: PLoS One. 2026 May 14;21(5):e0349099. doi: 10.1371/journal.pone.0349099 (PMC13175479; doi:10.1371/journal.pone.0349099)
Supplement: Attachment — Submitted filename: Response to Reviewers.docx [file pone.0349099.s002.docx]

The authors have substantially improved the manuscript in response to the previous review, particularly regarding the prophylactic framing of the animal model, data deposition for 16S rRNA sequencing, and additional analyses linking gastric microbiota to inflammatory markers. However, there remain several important issues that, in my view, still warrant a further round of revision before the manuscript can be considered for acceptance.

Major Comments

**1. Quantification and interpretation of gastric H. pylori burden**

The authors still do not provide a direct quantitative measure of gastric H. pylori burden (e.g., CFU counts or qPCR from gastric tissue), and rely instead on RUT results and relative abundance of Helicobacter from 16S rRNA amplicon sequencing as indirect indicators.

Given this limitation, the Discussion and Conclusion occasionally still use wording that implies a reduction in “load” or “colonization” in a way that suggests direct quantitative evidence. This overstates what the available data can support.

Request:

• Throughout the Discussion and Conclusion, I ask the authors to: Further soften and standardize the language so that claims are clearly limited to:

• reductions in Helicobacter relative abundance (from 16S data), and

• improvements in infection-related biomarkers (RUT, urease, PGI/PGII, histology, cytokines),

rather than “reducing H. pylori colonization” or “reducing bacterial load” per se.

• For example, expressions such as “reduced the load/colonization of H. pylori” should be systematically revised to formulations like “reduced Helicobacter relative abundance and infection-related markers”.

• In the Limitations section (or the final part of the Discussion), the authors should explicitly acknowledge that:

• Gastric H. pylori burden was not directly quantified using CFU counts or qPCR.

• The conclusions regarding anti–H. pylori effects therefore rely on RUT and relative abundance from 16S rRNA gene sequencing, rather than absolute bacterial load measurements.

This clarification is important to align the strength of the claims with the actual data and to transparently communicate methodological limitations to readers.

**2. Microbiota analysis – β-diversity methodology and PERMANOVA**

The authors state in their response that they have re-analysed β-diversity using PCoA on the Jaccard distance and performed PERMANOVA. However, the current manuscript text remains internally inconsistent and does not fully reflect these methodological changes.

**(a) PCA vs PCoA inconsistency**

In Methods (Section 2.5.3), β-diversity is still described as being analysed by PCA based on Jaccard (e.g., “PCA was employed to further analyze beta diversity”). In contrast, in Results (Section 3.4.3) and the relevant figure legend, the analysis is described as Principal Coordinate Analysis (PCoA) based on the binary Jaccard algorithm.

This discrepancy leaves it unclear what analysis was actually performed.

Request:

• Please ensure that the description of β-diversity analysis is fully consistent throughout the manuscript.

• If PCoA on the binary Jaccard distance was indeed used (which is methodologically appropriate), then Methods 2.5.3 must be revised to say PCoA (not PCA), and the language in all sections (Methods, Results, figure legends) should be harmonized accordingly.

**(b) PERMANOVA – description and reporting**

In the response letter, the authors state that they performed PERMANOVA with pairwise comparisons and FDR correction.

However, in Methods (Statistics, Section 2.6), there is no mention of PERMANOVA or any distance-matrix–based multivariate test. Only one-way ANOVA, Tukey’s post hoc test, and correlation heatmaps are described. In the Results and figure legends, there is no reporting of PERMANOVA outputs (no pseudo-F statistic, R², p-value, or number of permutations).

Thus, the manuscript does not currently support the claim that PERMANOVA was performed.

Request:

• If PERMANOVA has indeed been carried out:

• Add a clear description in Methods (Statistics) of:

• the distance matrix used (e.g., Jaccard),

• the PERMANOVA procedure (e.g., number of permutations),

• and how multiple testing was handled for pairwise comparisons (e.g., Benjamini–Hochberg).

• In the Results (and/or relevant figure legend), report at minimum the overall PERMANOVA result with pseudo-F, R², p-value, and number of permutations.

⸻

Minor Comments

**1. Taxonomic spelling and nomenclature**

Despite substantial improvement, several taxonomic spelling errors remain and should be corrected.

• In the Discussion: “Proteobactria” → “Proteobacteria”

• In the Abstract and Results: “unclassified-Muribaculaceaeae” → “unclassified-Muribaculaceae”

• In the Results: “Oscillospiracea”(Line 406) → “Oscillospiraceae”

**2. Other wording and typographical issues**

A few additional typographical and wording errors should be corrected for clarity and precision.

• Introduction (around line 83): “alleviating tail inflammation” should be corrected to “alleviating gastric inflammation”

• Methods (around lines 232–233): “Interleuki-8, Interleuki-1β, Interleuki-6” → “Interleukin-8, Interleukin-1β, Interleukin-6”

• Results (around line 371): “binary jaccard algorith”→ “binary Jaccard algorithm”

• Results (around line 364, IL-6 description)

The text currently states that the mean IL-6 concentration in the HY001 group is “higher than that of the HP group”, which contradicts the protective direction of effect described elsewhere and in the Discussion.

This is almost certainly a typo and should be corrected to “lower than that of the HP group”,

provided that the underlying data support this (if not, the interpretation in the Discussion should be adjusted accordingly).

• Figure 4 legend

The definitions of the HP and HY001 groups are currently confusing and partly duplicated, e.g.:

• “HP: treated by H. pylori SS1 group vs. pretreated by L. casei HY001 and treated by H. pylori SS1.”

• “HY001: treatment using L. case HY001 and treatment using H. pylori SS1; HP: treatment using H. pylori SS1.”

Please simplify and unify the legend.

• Figure 6 legend (gastric vs fecal)

In one place, the legend refers to the “composition of gastric microorganisms”, whereas in another location (around line 869) it refers to “composition of fecal microorganisms”. Since this study is based on gastric samples, all such references in the figure legends and text should be unified as “gastric microbiota” or “gastric microorganisms”, and any inadvertent “fecal” wording should be removed.
