# Supplementary material for: Multimodal anti-Helicobacter pylori effects of Lactobacillus casei HY001: Evidence from in vitro and in vivo studies
Source: PLoS One. 2026 May 14;21(5):e0349099. doi: 10.1371/journal.pone.0349099 (PMC13175479; doi:10.1371/journal.pone.0349099)
Supplement: Attachment — Submitted filename: Reply to reviewers 2.docx [file pone.0349099.s004.docx]

Dear reviewers:

Thank you very much for taking the time to review our manuscript entitled “Multimodal Anti-Helicobacter pylori Effects of Lactobacillus casei HY001: Evidence from In Vitro and In Vivo Studies” and for providing us with such thorough and insightful comments. We have carefully studied and thoroughly considered each of your points. Your suggestions are highly pertinent and valuable, and they have helped us to better recognize the shortcomings of our paper. We have revised the manuscript comprehensively and diligently in accordance with your recommendations.

Reviewer #1: This manuscript assesses anti–Helicobacter pylori properties of Lactobacillus casei HY001 using in-vitro assays and a mouse pretreatment model. The linkage between cellular readouts, histology/inflammation, and gastric microbiota is a strength. However, the central claim of “reduced colonization” is not supported by a direct quantitative gastric burden (CFU/qPCR). The prophylactic design is at times generalized to therapeutic benefit; the microbiota methods/terminology contain inconsistencies requires reinforcement; and nomenclature/English editing (e.g., Proteroidota) need correction. Data deposition for raw 16S reads is also recommended. In my view, the work is potentially publishable after major revision.

Major points
1. Quantification of gastric H. pylori burden is insufficient
Mouse infection status relies on rapid urease test, without CFU plating or qPCR of gastric tissue. RUT is semi-quantitative and not a gold-standard bacterial load measure. Please add a direct quantitative readout of gastric colonization—such as CFU counts and/or qPCR from gastric tissue.
Across the Discussion/Conclusion you state that HY001 effectively reduced colonization, yet no direct quantitative burden (gastric CFU or qPCR) is reported. Without such metrics, please soften the language (e.g., “associated with reduced markers of infection”) or add the missing quantification.

**Response:** We sincerely thank the reviewer for this insightful comment and for highlighting the importance of quantitative assessment of *H. pylori* burden. We agree that CFU plating or qPCR would provide a direct quantitative measure of bacterial load.

Please allow us to explain the attempts we made during the experimental process: We initially tried to perform CFU plate counts for *Helicobacter pylori* in mouse gastric tissue using BHI + defibrinated sheep blood medium, hoping to obtain direct quantitative data. However, due to the complex inherent microbiota in the mouse stomach, the process was significantly interfered with by the growth of other microorganisms, preventing us from obtaining accurate and specific colony count data for *H. pylori*.

It was precisely due to this technical challenge that we adopted 16S rRNA gene amplicon sequencing of the total gastric microbiota as a solution. While this method differs from absolute quantitative measures like CFU or qPCR, it effectively circumvents interference from other microbes and provides us with a specific and reliable indirect indicator for assessing the impact of HY001 treatment on the survival state of *H. Pylori* within the gastric environment.

While 16S rRNA gene sequencing is primarily used for relative abundance analysis, the data on the relative abundance of the *Helicobacter* genus serves as a strong and complementary indicator of the changes in *H. pylori* load within the gastric environment.

As the reviewer can find in our results section (Line 404-408), the 16S rRNA gene sequencing data clearly demonstrated that the administration of HY001 significantly reduced the relative abundance of the *Helicobacter* genus in the gastric tissue compared to the infected control group. This finding, consistent with the RUT results, provides robust evidence that HY001 treatment effectively suppressed *H. pylori* colonization.

However, we fully acknowledge the reviewer's point that our original language in the Discussion and Conclusion sections may have been too strong given the methodological approach. Therefore, we have followed the reviewer's excellent suggestion and have revised the relevant statements throughout the manuscript to soften our language. We have replaced phrases like "effectively reduced colonization" with more precise descriptions such as " *L. casei HY001* effectively reduced the indicators related to *Helicobacter pylori* infection ".

We believe that the combination of the RUT results and the 16S rRNA sequencing data, together with the toned-down language, now presents a more accurate and convincing argument for the efficacy of HY001 against *H. pylori*. We hope that these clarifications and textual revisions adequately address the reviewer's concern.

2. Animal study design demonstrates prophylaxis, not treatment
HY001 was given before and through infection, demonstrating pretreatment/prophylactic effects rather than therapy of established infection; claims drift toward “therapeutic” benefit. Please align Abstract/Discussion/Conclusion phrasing accordingly and avoid general therapeutic claims such as “foundation for an anti-H. pylori drug” unless a post-infection arm is added.
If you want to discuss a therapeutic effect, please include a post-infection treatment arm in which HY001 is initiated after HP colonization is established.

**Response:** We sincerely thank the reviewer for this crucial observation. We completely agree that our experimental design, where in HY001 administration began prior to infection, is indeed a model of prophylaxis and not therapy of an established infection. We apologize for any overinterpretation in our original phrasing that may have suggested otherwise. As suggested, we have thoroughly revised the manuscript to ensure all language accurately reflects the prophylactic nature of our study design. The claims have been carefully aligned throughout the text. We believe these revisions have fully addressed your concern by ensuring our claims are strictly aligned with our experimental design. We are grateful for this insightful comment, which has greatly improved the clarity and accuracy of our manuscript. Thank you again for your time and valuable feedback.

The specific changes made in the manuscript are as follows:

Abstract Section (Line 29,) Changed "…HY001 treatment. "to"…HY001 intervention."

Discussion Section (Line 528-529): Changed "suggesting that HY001 mitigated gastric inflammation in mice with H. pylori-induced gastritis." to "suggesting that the HY001 intervention mitigated gastric inflammation caused by *Helicobacter pylori* in mice. "

Conclusion Section (Line 587-588): Changed: " These provide a theoretical foundation for its application as an anti- H. pylori drug. "to" These provide a theoretical basis for the application of this probiotic in alleviating Helicobacter pylori infection. "

We hope that these clarifications and textual revisions adequately address the reviewer's concern.

3. Microbiota analysis: terminology accuracy and method modernity
Across the text, figures, and legends, phylum-level names are inconsistently reported using a mixture of legacy and updated nomenclature. Because terminological consistency is essential for clarity and reproducibility, please apply it uniformly throughout. Concretely, we see both Bacteroidetes vs. Bacteroidota and Proteobacteria vs. Proteroidota (The manuscript uses “Proteroidota,” which is not a recognized phylum name in major taxonomic frameworks and appears to be a typographical error. It likely intends Pseudomonadota).
The manuscript states “Principal Component Analysis (PCA) based on the binary Jaccard algorithm,” which is methodologically inconsistent: Jaccard is a non-Euclidean distance, whereas PCA is defined in Euclidean (covariance) space. For β-diversity ordination you should use Principal Coordinates Analysis (PCoA) on the Jaccard distance (or NMDS), rather than PCA.
In addition, no statistical test of between-group differences is reported. Please provide a PERMANOVA with the pseudo-F, R², p-value, number of permutations, and apply multiple-testing correction (e.g., Benjamini–Hochberg) for pairwise contrasts when applicable.

Response: We are deeply grateful to the reviewer for their meticulous review and for providing these critical and constructive comments. They have significantly helped us improve the accuracy and rigor of our microbiota analysis. We have addressed each point as follows:

**1. Terminology Accuracy:**
We sincerely apologize for the inconsistencies and errors in the phylum-level nomenclature. The reviewer is absolutely correct. Action Taken: We have thoroughly reviewed the entire manuscript, including the main text, figures, and figure legends, to ensure uniform use of the updated, modern phylum names as per the current taxonomic framework. Specifically: *Bacteroidetes* has been uniformly changed to *Bacteroidota*.

**2. Methodological Consistency for β-diversity Analysis:**
The reviewer is entirely right to point out the methodological inconsistency. PCA is not suitable for Jaccard distances. Action Taken: We have re-analyzed our β-diversity data using the correct method. We have replaced the original PCA plot with a Principal Coordinates Analysis (PCoA) plot based on the binary Jaccard distance matrix. The corresponding text in the Methods and Results sections, as well as the Figure legend, have been updated accordingly. All mentions of "PCA" in this context have been changed to "PCoA".

**3. Statistical Testing for β-diversity:**
We agree that the lack of statistical testing for the β-diversity results was a major omission. Action Taken: We have now performed the recommended statistical analysis. We conducted a Permutational Multivariate Analysis of Variance (PERMANOVA) with 9999 permutations on the Jaccard distance matrix to test for overall group differences. For significant overall effects, we performed post-hoc pairwise PERMANOVA tests with Benjamini-Hochberg (FDR) correction for multiple comparisons. The results of these analyses (pseudo-F statistic, R² value, p-value, and the number of permutations) have now been added to the Results section and the corresponding Figure legend.

4. 16S data transparency and availability
Methods list the 16S pipeline, but no repository accessions are provided; the Data Availability statement currently points to “within the manuscript,” which is insufficient for sequencing data. Please deposit raw reads and provide accession IDs.

Thank you for this important reminder regarding data transparency. We agree that the public deposition of raw sequencing data is essential.

In response to this comment, we have now deposited the raw 16S rRNA gene sequencing reads to the NCBI Sequence Read Archive (SRA). The data is currently under processing and will be publicly accessible upon publication. The associated BioProject accession number is PRJNA1358791. We have updated the Data Availability Statement in the manuscript to clearly state the accession information. The statement now reads:

"The raw 16S rRNA gene sequencing data generated in this study have been deposited in the NCBI SRA under BioProject accession number PRJNA1358791. All other data supporting the findings of this study are available within the article and its supplementary information. "We will also ensure that the accession number is included in the Methods section where the 16S sequencing pipeline is described. We appreciate the reviewer's diligence in ensuring the completeness and reproducibility of our work.

Minor points
•Abstract scope drift: The latter half of Abstract lists multiple taxa (phylum/family/genus), which dilutes focus. In an abstract, avoid laundry lists; instead, state that the gastric microbiota composition shifted significantly. Leave taxon-level details to the Results.

**Response:** We thank the reviewer for this valuable suggestion to improve the clarity and focus of our Abstract. We agree that listing numerous taxonomic names in the Abstract can be distracting and dilute the key messages. In response, we have revised the latter part of the Abstract accordingly. We have removed the detailed list of specific phyla, families, and genera. Instead, we now succinctly state the core finding that the composition of the gastric microbiota was significantly altered by HY001 treatment, and we refer readers to the Results section for in-depth taxonomic details.

•Figure legend scope: Some legends refer to “fecal microorganisms,” but the study focuses on gastric samples—please correct to “gastric.”

**Response:** We sincerely thank the reviewer for their meticulous attention to detail. We apologize for this oversight in our figure legends. The reviewer is absolutely correct; our study analyzed gastric microbiota, not fecal. We have carefully reviewed all figure legends (and the main text) and have corrected any erroneous mentions of "fecal" to "gastric" to accurately reflect the sample source. Specifically, this correction has been made in the legends for [Figure 6 (a)]. We appreciate the reviewer for helping us improve the accuracy and clarity of our manuscript

•Taxonomic spellings: Correct misspellings such as “Protebacteria → Proteobacteria,” “Muribaculacea → Muribaculaceae,” “Lachnosoiraceae → Lachnospiraceae.” “Protebacteria” also appears in Figure 7.

**Response:** We sincerely thank the reviewer for their meticulous proofreading and for identifying these typographical errors in taxonomic names. We apologize for these oversights.

We have carefully corrected all the misspellings pointed out by the reviewer throughout the manuscript and in the figures. Specifically: “Protebacteria” has been corrected to “Proteobacteria” in the text and in Figure 7. “Muribaculacea” has been corrected to “Muribaculaceae”. “Lachnosoiraceae” has been corrected to “Lachnospiraceae”. Furthermore, to ensure the highest level of accuracy, we have conducted a comprehensive check of the entire manuscript (including the main text, figures, and supplementary materials) to identify and correct any other potential spelling errors in taxonomic nomenclature. We are grateful to the reviewer for helping us enhance the quality and precision of our work.

•Method citations: Ensure references in Methods directly support the techniques used (e.g., ELISA procedures, microbiome pipelines) and remove unrelated citations.

**Response:** Thank you for this valuable comment. We have thoroughly reviewed all citations in the methods section to ensure each one directly and accurately supports the specific techniques described. The corresponding revisions have been made to the manuscript, as detailed below:

**Regarding the ELISA procedure:** We have revised the manuscript (Line 175-178) to specify the manufacturer (Bioswamp, Wuhan, China) of the ELISA kit used. The use of this specific kit and the general procedure are directly supported by Reference 30-32, which employed the same commercial assay. In the section of determining inflammatory factors through animal experiments (Line 232-235): We have also added relevant references. The use of this specific kit and the general procedure are directly supported by Reference 33-34, which employed the same commercial assay. Therefore, we have retained and strengthened this citation to directly support our experimental approach.

Regarding the microbiome analysis pipeline: We have clarified in the text (Line 249-254) that our microbial community profiling was performed using the the BMK Cloud platform for sequencing the 16S rRNA gene V3-V4 region, and that our bioinformatic processing (using QIIME2 with the SILVA database) directly followed the workflow established in Reference 36-38. Thus, we maintain that Reference 36-38 is the appropriate and direct citation for our analytical platform and pipeline.

**Regarding other technical methods:** We have performed a comprehensive check of all citations in the Methods section. Every remaining citation now accurately and directly underpins its corresponding experimental technique.

We believe these revisions have significantly improved the accuracy and rigor of the Methods section by ensuring all references provide direct methodological support. Thank you again for helping us improve our manuscript.

Reviewer #2:

**Materials and methods**
1, Prior to screening, the selection criteria for the six Lactobacillus casei strains are not clearly mentioned beyond their isolation from sour milk. It would be better if authors give rationale for why these specific strains were chosen or their genomic/phenotypic differences.

**Response:** The six *L. casei* strains were randomly selected from a larger collection of isolates that we obtained from several independent batches of sour milk. The rationale for this approach was to avoid any pre-selection bias and to obtain a sample of the natural *L. casei* found in this environment. While we acknowledge that a pre-screening based on genomic or phenotypic markers would be valuable, we believe that this random selection from diverse sources provides a robust and unbiased initial comparison of functional properties, which is the core objective of this study. We have revised the 'Materials and Methods' section to better explain this selection strategy.

2, Authors have used only one gastric cell line in their in vitro adhesion assays. What would be the justification for reliance on a single cell line? It would limit the generalizability of adhesion and displacement results.

**Response:** We sincerely thank the reviewer for this critical comment, which helps to clarify the scope and context of our findings. We acknowledge that the use of a single cell line is a limitation for the broad generalizability of our results.

In this study, we selected the AGS cell line as our in vitro model for two primary reasons. It is a mature and widely used model in the field of gastric microbiology, especially suitable for studying the interaction between bacteria such as Lactobacillus for studying [1, 2]. For instance, numerous studies have utilized this model to reveal the role of Lactobacillus in inhibiting pathogen colonization, regulating host immune responses, and protecting the integrity of the epithelial barrier [2, 3]. The AGS model provides a highly reproducible and standardized system ideal for this specific comparative objective. This choice enables meaningful comparisons with a large body of existing research results. Secondly, the core objective of our adhesion experiments is to confirm in a simplified model whether the "competitive adhesion" effect holds true under the same and controlled conditions. Provide targets and basis for future research: The results of this study will provide crucial theoretical support and effective candidate strains for subsequent animal experiments.

We believe that this clarification strengthens the manuscript and provides a balanced interpretation of our data.

References are listed below:

[1] Kim, J. M., et al. (2003). Differential expression of gastric mucin 2 and interleukin-8 in Helicobacter pylori-infected gastric ulcer patients. *The Korean Journal of Internal Medicine*, 18(1), 7–13.

[2] Wang, Z. H., et al. (2013). Meta-analysis of the efficacy and safety of Lactobacillus-containing and Bifidobacterium-containing probiotic compound preparation in Helicobacter pylori eradication therapy. *Journal of Clinical Gastroenterology*, 47(1), 25-32.

[3] Yang, Y. J., & Chuang, C. C. (2017). *Lactobacillus plantarum* attenuates *Helicobacter pylori*-induced gastritis by restoring the gastric microbiota and strengthening the epithelial barrier. *Frontiers in Microbiology*, 8, 2233.

3, Authors have used on female mice as animal models. The gender differences in studying immune response and microbiota composition could influence results. Therefore, it would be better if both sexes are included in the study.

**Response:** We thank the reviewer for this insightful and highly relevant comment. We fully acknowledge that sex differences can significantly influence immune responses and microbiota composition, and we agree that including both male and female animals in a study generally provides more comprehensive and translatable findings.

Our decision to use female mice in this initial study was not made lightly but was based on well-established methodological considerations specific to the *Helicobacter pylori* mouse model, which we believed would provide the most robust foundation for our primary research question.

The primary justification is rooted in the established literature on *H. pylori* colonization in mice. Several key studies have demonstrated that female mice exhibit superior and more reliable gastric colonization by *H. pylori* compared to males. For instance, a study by Court M et al**,** 2003, J Pathol showed that female mice developed more severe and earlier-onset gastric pathology from *H. pylori* infection than males, with significantly greater chronic inflammation in the long term. Following *H. pylori* infection, female mice exhibited an earlier onset of gastric disease and a trend toward more severe pathology compared to males, a difference that was significant for chronic inflammation in long-term infections.

This phenomenon has been attributed to factors including sex hormone differences, which can modulate gastric physiology and immune responses. By using female mice, we followed a common and optimized protocol to maximize the consistency and success rate of infection, thereby reducing the potential for failed colonization as a confounding variable and strengthening the internal validity of our intervention study.

We recognize that this choice represents a limitation in the generalizability of our specific results to male mice. In direct response to the reviewer's valuable point, we have now revised our manuscript to explicitly address this limitation. Accordingly, we have added a new sentence in the concluding paragraph of the Discussion to explicitly state this limitation and our future direction. The added text lines 575-579 reads:

“Finally, it should be noted that this study utilized only female mice. While this was necessary to establish a consistent model for the initial validation of HY001's efficacy, it limits the generalizability of our findings. Future studies employing both male and female animals are essential to determine whether the therapeutic effects of HY001 are sex-dependent.”

We believe that these proofs clearly explain the purpose of our use of female mice, and at the same time demonstrate that our model selection is reasonable in accordance with the specific requirements of the Helicobacter pylori research. Once again, we thank you for your constructive suggestions, which undoubtedly enhance the clarity and academic rigor of our manuscript. References are listed below:

Court M, Robinson PA, Dixon MF, Jeremy AH, Crabtree JE. The effect of gender on Helicobacter felis-mediated gastritis, epithelial cell proliferation, and apoptosis in the mouse model. J Pathol. 2003 Oct; 201 (2):303-11. doi: [10.1002/ path. 1422](https://doi.org/10.1002/path.1422). PMID: 14517848.

3, The methodology for the formulation of L. casei is not explained. Authors should describe explicitly the inclusion of some appropriate negative or vehicle controls.

Response: We thank the reviewer for this insightful comment. We agree that clarifying the probiotic formulation and the control groups is essential for the reproducibility and interpretation of our study. We have now added detailed descriptions in the revised manuscript, specifically in the Materials and methods section (Animal and experimental design) under new subsection titled "Probiotic and helicobacter pylori preparation "and " Experimental design and animal groups"

The changes are as follows:

Part1 Probiotic and helicobacter pylori preparation

*L. casei* HY001 bacterium suspension preparation: The strain of *L. casei* HY001 was transferred to the liquid MRS medium, and was cultured for 16 hours. The bacterial cells were then harvested by centrifugation at 8,000 × rpm for 3 minutes, washed twice with 0.9% Sodium Chloride Injection, and the bacterial concentration was adjusted with 0.9% Sodium Chloride Injection to deliver a final dose of 5×10^9^ CFU/mL.

*H. pylori* SS1 bacterium suspension preparation: were incubated in improved Columbia blood solid media (BHI), under microaerophilic conditions (5% O_2_, 10% CO_2_, 85% N_2_) at 37 ℃ for 48-72 h. *H. pylori* SS1 cells were collected, washed twice with 0.9% Sodium Chloride Injection, and then the bacterial concentrations were adjusted to 8×10^9^ CFU/mL using 0.9% Sodium Chloride Injection to obtain *H. pylori* SS1 suspensions.

Part 2 Experimental design and animal groups

(1) the Control group (NC group), which received only a standard diet and gavaged with 300 μ L 0.9% Sodium Chloride Injection throughout the entire testing period; After pre-treatment with 0.9% Sodium Chloride Injection for one week, mice were gavaged with 300 μ L 0.9% Sodium Chloride Injection every two days for a total of 7 times.

(2) the model group (HP group), which received only a standard diet and gavaged with 300 μ L 0.9% Sodium Chloride Injection throughout the entire testing period; After pre-treatment with 0.9% Sodium Chloride Injection for one week, mice were gavaged with 300 μ L H. pylori SS1 suspensions every two days for a total of 7 times.

(3) the *L. casei* HY001 intervention group (HY001 group), which received only a standard diet and gavaged with 300 μ L *L. casei* HY001 bacterium suspension throughout the entire testing period; After pre-treatment with 0.9% Sodium Chloride Injection for one week, mice were gavaged with 300 μ L *H. pylori* SS1 suspensions every two days for a total of 7 times.

We believe these clarifications significantly strengthen the methodology of our paper and thank the reviewer for the suggestion.

4, The data of some cytokine is showing nonsignificant trends but authors have discussed them as biologically relevant without sufficient statistics.

**Response:** We thank the reviewer for this critical observation. We agree that the p-values for certain cytokines, such as IL-6, did not reach the conventional threshold of statistical significance (p < 0.05). However, we believe it is scientifically valuable to discuss these trends for the following reasons:

Biological plausibility: The observed trend for IL-6 is directionally consistent with our central hypothesis that H. pylori infection induces gastritis in mice. As a key pro-inflammatory cytokine, the intervention of HY001 led to a decrease in IL-6. Even though it was only a slight trend, it was consistent with the overall mechanism we proposed.

Consistency with other significant findings: This trend forms a coherent biological narrative with our other statistically significant results. For instance, we observed a significant the intervention of HY001 led to decrease in pro-inflammatory cytokines like IL-8, IL-1β, and TNF-α, (p < 0.01) and a significant improvement in pathological examination and immunohistochemistry. In this context, the upward trend in the proinflammatory IL-6 provides a more complete picture of the immune response.

**Changes made in the manuscript:**

To address the reviewer's concern directly and ensure appropriate scientific rigor, we have revised the text to more cautiously describe these findings:

In the Discussion section (Line 489-491)，we have added the following clarification:

" Although the decrease in IL-6 did not reach statistical significance, the observed trend is directionally consistent with the reduction in inflammatory factors and our proposed mechanism."

We believe these revisions strike a balance between scientific caution and the discussion of biologically relevant patterns that could inform future research. Thank you for prompting us to improve the clarity and precision of our manuscript.

**Results**
1, It would be better if authors give clinical relevance of their findings of in vitro and animal models. Authors have studied microbiota changes but did not establish functional causality between specific microbial shifts and observation of anti-inflammatory or anti-*H. pylori* effects.

**Response:** We sincerely thank the reviewer for this valuable suggestion. In response, we have performed a targeted correlation analysis to establish a functional link between the specific microbial shift induced by *L. casei* HY001 treatment and the observed anti-inflammatory effects in our animal model.

Our analysis revealed a critical and specific association: the genus *Helicobacter* demonstrated a significant positive correlation with key pro-inflammatory factors. This finding provides a plausible mechanistic link. We propose that the therapeutic effect of *L. casei* HY001 is mediated, at least in part, through the suppression of this pro-inflammatory taxon, *Helicobacter*. The reduction in *Helicobacter* abundance, as a result of HY001 intervention, directly corresponds to the alleviation of gastric inflammation observed in our mouse model. This establishes a functional causality between a specific microbial shift and the anti-inflammatory outcome.

We believe these new analyses and discussions directly address the reviewer's concern by establishing a functional bridge between the specific microbial shifts and the therapeutic outcomes, thereby enhancing the clinical translatability of our work.

2, Authors should critically discuss the contradiction of conflicting microbiota results for their increased abundance of genera such as Helicobacter in the treatment group.

**Response:** We thank the reviewer for this critical comment. We acknowledge that the original text contained a contradictory statement regarding the microbiota results, which was due to an error in writing. In response, we have taken the following action in the revised manuscript: We have corrected the incorrect statements. in the result section

3, Authors have not addressed the potential for probiotics causing adverse effects, especially with long-term administration.

**Response:** We thank the reviewer for raising this critical point regarding the safety profile of probiotic administration, which is indeed an essential aspect of any intervention study. We agree that discussing potential adverse effects is crucial for a complete and balanced scientific narrative. In response to this comment, we have now included a dedicated section in the revised manuscript to address this issue comprehensively.

Our response is based on the following three pillars:

**Study-Specific Safety Monitoring:** Throughout the defined period of our study 4 weeks, we actively monitored all mice for any immediate adverse effects. We did not observe any adverse events (such as "severe diarrhea, vomiting, bloating, or infections") related to the intervention of *L. casei* HY001. This statement has been added to the **Results section.**

We acknowledge that our study was not primarily powered or designed to detect rare or very long-term adverse effects. Therefore, in the **Discussion section,** we have now added a paragraph that explicitly states:

"While no adverse events were observed in our cohort during the intervention period, and the existing literature supports the safety of the probiotic strains employed, it is important to note that our study was limited in its duration and scale for a comprehensive safety assessment. Future large-scale, long-term epidemiological studies are warranted to fully elucidate the safety profile of probiotic supplementation over extended periods."

We believe these additions significantly strengthen the manuscript by proactively addressing the important issue of probiotic safety, thereby enhancing the transparency and clinical relevance of our work. We thank the reviewer again for this valuable suggestion.

**Discussion**
1, Authors have merely discussed the recent development and contradictory findings in probiotic therapy against H. pylori. Authors should add comparative discussion on why HY001 is superior or mechanistically different.

**Response:** We sincerely thank the reviewer for this insightful and critical comment. We agree that a comparative discussion highlighting the potential superiority and mechanistic distinctions of our specific strain*, Lactobacillus casei* HY001, is essential to strengthen the impact of our manuscript.

we have thoroughly revised the discussion section to incorporate a dedicated paragraph that explicitly addresses this point. The new text provides a comparative perspective on why HY001 may be superior and elaborates on its unique, multi-faceted mechanism of action compared to other commonly studied probiotic strains.

The key points we have now emphasized are as follows:

**Novel Direct Inhibition via Urease Suppression:** A key mechanistic differentiator of HY001 is its ability to produce metabolites that directly inhibit the urease activity of H. pylori. Urease is a crucial virulence factor for *H. pylori* colonization and survival in the acidic stomach. strategy that is not commonly reported for all probiotic strains, going beyond simple acid production.

**Effective Competitive Exclusion:** Our data confirm that HY001 effectively competes with *H. pylori* for adhesion sites on gastric epithelial cells. This physical blockade prevents the initial attachment and colonization of the pathogen, serving as a critical first line of defense.

**Targeted Anti-inflammatory Action:** HY001 demonstrates a potent immunomodulatory effect by significantly suppressing the NF-κB signaling pathway. This specific action directly counteracts the primary mechanism by which *H. pylori* induces gastritis, leading to a marked reduction in the production of pro-inflammatory cytokines like IL-8. This targeted approach to mitigating inflammation addresses a core aspect of *H. pylori*-related pathology.

**Restoration of Gastric Microecological Homeostasis:** Moving beyond direct pathogen interaction, HY001 exerts a broader, systemic effect on the gastric microenvironment. Our analysis shows that administration of HY001 significantly increases microbial species richness and diversity, suppresses the relative abundance of harmful bacteria, and promotes the growth of beneficial bacteria. This ability to restore a healthy microecological balance represents a superior, holistic approach to therapy, helping to create an environment that is inherently resistant to *H. pylori* colonization and pathogenesis.

In summary, the revised discussion clearly argues that HY001's potential superiority stems from its comprehensive, multi-level strategy. It acts not only directly on the pathogen (via urease inhibition and competitive exclusion) and on the host's inflammatory response (via NF-κB suppression), but also on the broader microbial community to restore a healthy gastric ecosystem. This synergistic combination of mechanisms differentiates HY001 from probiotics with more limited modes of action and positions it as a highly promising candidate for combating *H. pylori* infection.

We are grateful for the reviewer's suggestion, which has undoubtedly helped us improve the clarity and significance of our manuscript. The changes are marked in the revised manuscript, and we hope the new discussion satisfactorily addresses the comment.

**Writing and Presentation**
1, The manuscript has grammatical errors, awkward phrasing, and redundancy.

**Response:** We sincerely thank the reviewer for pointing this out. We have thoroughly reviewed the entire manuscript and made comprehensive revisions to address the issues of grammar, phrasing, and redundancy.

Specifically, we have taken the following actions:

Language Polishing: The manuscript has been carefully edited to correct all identified grammatical errors and to improve sentence fluency and clarity. Phrasing Improvement: Awkward or unclear phrases have been rephrased for better readability and precision of scientific expression. Elimination of Redundancy**:** Repetitive statements and redundant information have been removed to make the text more concise and focused.

We believe the manuscript is now greatly improved in terms of language quality and clarity. The changes have been made throughout the manuscript and are marked in the revised version using the "Track Changes" function for your convenience.

2, Need corrections for some terms such as anaplasmosis portal and thick-walled portal which are related to microbiota analysis. These are unclear or not correctly used.

**Response:** We sincerely thank the reviewer for pointing out these inaccurate and unclear terminologies. We apologize for the errors. The terms "anaplasmosis portal" and "thick-walled portal" were indeed used incorrectly and do not properly describe concepts in microbiota analysis.

As the reviewer suggested, we have thoroughly reviewed the manuscript and have removed or replaced these incorrect terms with accurate and standard microbiological terminology throughout the text. The relevant sections in the Results and Methods have been revised for clarity and precision.

We are grateful for the reviewer's expertise, which has helped us improve the quality and readability of our manuscript.

3, The abbreviations are inconsistently used in different sections, which are confusing.

**Response:** We thank the reviewer for this helpful comment. We apologize for the inconsistency in the use of abbreviations, which has now been thoroughly addressed in the revised manuscript.
Specifically, we have taken the following steps to ensure consistency and clarity: We have conducted a systematic check of all abbreviations used throughout the entire manuscript. We have ensured that each term is defined upon its first use in both the abstract and the main text. We have verified that after being defined, each abbreviation is used consistently in its shortened form throughout the text, including in figures and tables. We believe these measures have resolved the inconsistency and significantly improved the clarity of the manuscript.

4, Authors should strengthen experimental design by including additional gastric cell lines and/or both sexes in animal studies or provide strong justification for their choices.

**Response:** We sincerely thank the reviewer for this critical comment, This issue has been addressed in the second and third questions of the materials and methods section of Reviewer #2's submission.

5, Authors should provide more robust statistical reporting and improve figure legends for independent comprehension.

**Response:** We sincerely thank the reviewer for this valuable feedback. We agree that robust statistical reporting and clear figure legends are crucial for the reader's understanding. We have thoroughly revised the manuscript to address this point, implementing more detailed statistical reporting and significantly enhancing the figure legends. The specific changes are detailed below.

**Enhancements to Statistical Reporting**

We have revised our statistical reporting to be more rigorous and transparent, specifically for all group comparisons.

**Clarification of Statistical Methods:** For multiple group comparisons meeting assumptions of normality and homogeneity of variances, we used one-way ANOVA. To identify specific differences between groups, we performed post-hoc tests.

Significance Labeling (Key Improvement): We have adopted the standard" lettering scheme" to denote statistical significance across groups. The rule is as follows: Groups that share a common lowercase letter (a, b, c,...) are not statistically significantly different from each other (P ≥ 0.05). Groups that do not share a common letter are statistically significantly different (P < 0.05).

We believe the revisions made to the statistical reporting and figure legends have significantly improved the clarity and robustness of our manuscript. The figures can now be comprehended independently, and the statistical analysis is reported with greater transparency.

We hope these changes meet with the reviewer's approval.

Sincerely,
Zhonghua Lv
Harbin Pharmaceutical Group Bioengineering Co., LTD.
