# Supplementary material for: Multimodal anti-Helicobacter pylori effects of Lactobacillus casei HY001: Evidence from in vitro and in vivo studies
Source: PLoS One. 2026 May 14;21(5):e0349099. doi: 10.1371/journal.pone.0349099 (PMC13175479; doi:10.1371/journal.pone.0349099)
Supplement: Attachment — Submitted filename: Replay reviewers.docx [file pone.0349099.s005.docx]

The authors have substantially improved the manuscript in response to the previous review, particularly regarding the prophylactic framing of the animal model, data deposition for 16S rRNA sequencing, and additional analyses linking gastric microbiota to inflammatory markers. However, there remain several important issues that, in my view, still warrant a further round of revision before the manuscript can be considered for acceptance.

Major Comments

**1. Quantification and interpretation of gastric H. pylori burden**

The authors still do not provide a direct quantitative measure of gastric H. pylori burden (e.g., CFU counts or qPCR from gastric tissue), and rely instead on RUT results and relative abundance of Helicobacter from 16S rRNA amplicon sequencing as indirect indicators.

Given this limitation, the Discussion and Conclusion occasionally still use wording that implies a reduction in “load” or “colonization” in a way that suggests direct quantitative evidence. This overstates what the available data can support.

Request:

• Throughout the Discussion and Conclusion, I ask the authors to: Further soften and standardize the language so that claims are clearly limited to:

• reductions in Helicobacter relative abundance (from 16S data), and

• improvements in infection-related biomarkers (RUT, urease, PGI/PGII, histology, cytokines),

rather than “reducing H. pylori colonization” or “reducing bacterial load” per se.

• For example, expressions such as “reduced the load/colonization of H. pylori” should be systematically revised to formulations like “reduced Helicobacter relative abundance and infection-related markers”.

• In the Limitations section (or the final part of the Discussion), the authors should explicitly acknowledge that:

• Gastric H. pylori burden was not directly quantified using CFU counts or qPCR.

• The conclusions regarding anti–H. pylori effects therefore rely on RUT and relative abundance from 16S rRNA gene sequencing, rather than absolute bacterial load measurements.

This clarification is important to align the strength of the claims with the actual data and to transparently communicate methodological limitations to readers.

**Response:** Thank you for this critical and constructive feedback. We fully agree that the language used to describe the effects on *H. pylori* must precisely reflect the indirect measures employed in our study (RUT and 16 S rRNA gene sequencing relative abundance) and avoid any unintended implication of direct quantitative load measurement (e.g., via CFU or qPCR). We have carefully revised the manuscript accordingly to ensure the strength of our claims is perfectly aligned with the data.

**Systematic Revision of Language in the Discussion and Conclusion:**
We have performed a thorough review of the entire manuscript, with particular focus on the Discussion and Conclusion sections. All phrases that could be interpreted as claiming a reduction in absolute bacterial "load" or "colonization" have been revised. The language now consistently frames our findings based on the available evidence.

**Strengthened Methodological Limitation Statement:**
We have significantly expanded the Limitations section to include a dedicated, explicit acknowledgment as suggested. This statement now clearly:

Declares the lack of direct quantitative measures (CFU/qPCR).

Explicitly states that all anti-*H. pylori* conclusions are derived from RUT and 16 S rRNA gene sequencing relative abundance data.

Guides the reader to interpret our findings within this specific methodological context.

**Added text in the Limitations section (Final part of the Discussion):**

**Methodological Note on H. pylori Assessment:** It is important to explicitly acknowledge that this study did not employ direct quantitative methods, such as colony-forming unit (CFU) counts or quantitative PCR, to measure the absolute gastric *H. pylori* load. Therefore, our conclusions regarding the anti-*H. pylori* effects are based solely on (1) outcomes from the rapid urease test (RUT), and (2) changes in the relative abundance of the *Helicobacter* genus as detected by 16 S rRNA gene sequencing. Throughout this manuscript, descriptions of "reducing" or "suppressing" *H. pylori* refer specifically to these indirect indicators of infection presence and activity, not to a measured decrease in absolute bacterial count. Future studies incorporating direct quantitative techniques will be valuable to confirm these observations.

We believe these comprehensive revisions have successfully addressed your concern. The manuscript now more accurately and transparently communicates the nature of our evidence, ensuring that readers can appropriately evaluate the findings. Thank you again for your valuable guidance in improving the precision and rigor of our work.

**2. Microbiota analysis – β-diversity methodology and PERMANOVA**

The authors state in their response that they have re-analysed β-diversity using PCoA on the Jaccard distance and performed PERMANOVA. However, the current manuscript text remains internally inconsistent and does not fully reflect these methodological changes.

**(a) PCA vs PCoA inconsistency**

In Methods (Section 2.5.3), β-diversity is still described as being analysed by PCA based on Jaccard (e.g., “PCA was employed to further analyze beta diversity”). In contrast, in Results (Section 3.4.3) and the relevant figure legend, the analysis is described as Principal Coordinate Analysis (PCoA) based on the binary Jaccard algorithm.

This discrepancy leaves it unclear what analysis was actually performed.

Request:

• Please ensure that the description of β-diversity analysis is fully consistent throughout the manuscript.

• If PCoA on the binary Jaccard distance was indeed used (which is methodologically appropriate), then Methods 2.5.3 must be revised to say PCoA (not PCA), and the language in all sections (Methods, Results, figure legends) should be harmonized accordingly.

**(b) PERMANOVA – description and reporting**

In the response letter, the authors state that they performed PERMANOVA with pairwise comparisons and FDR correction.

However, in Methods (Statistics, Section 2.6), there is no mention of PERMANOVA or any distance-matrix–based multivariate test. Only one-way ANOVA, Tukey’s post hoc test, and correlation heatmaps are described. In the Results and figure legends, there is no reporting of PERMANOVA outputs (no pseudo-F statistic, R², p-value, or number of permutations).

Thus, the manuscript does not currently support the claim that PERMANOVA was performed.

Request:

• If PERMANOVA has indeed been carried out:

• Add a clear description in Methods (Statistics) of:

• the distance matrix used (e.g., Jaccard),

• the PERMANOVA procedure (e.g., number of permutations),

• and how multiple testing was handled for pairwise comparisons (e.g., Benjamini–Hochberg).

• In the Results (and/or relevant figure legend), report at minimum the overall PERMANOVA result with pseudo-F, R², p-value, and number of permutations.

**Response to Reviewer Comment:**

Thank you for this detailed and crucial observation regarding the methodological description of our β-diversity and PERMANOVA analyses. We sincerely apologize for the significant discrepancy between our response letter and the manuscript text, and for the resulting confusion. You are absolutely correct, and we have now comprehensively revised the manuscript to ensure complete consistency and transparent reporting.

The following changes have been made to address both points:

(a) Clarification and Harmonization of PCoA Methodology:

**In the Methods (Section 2.5.3, Line 267-268, page 14):** The text has been revised to accurately describe the analysis performed. It now reads: "Principal Coordinate Analysis (PCoA) based on the binary Jaccard distance matrix was employed to visualize β-diversity..."

**In the Results (Section 3.4.3, Line 376-384，page 19) and Figure Legends:** We have verified that all references to this analysis now consistently use "Principal Coordinate Analysis (PCoA)".

This change eliminates the previous "PCA vs. PCoA" inconsistency and correctly identifies the distance-based ordination method used.

**(b) Full Integration and Reporting of PERMANOVA:**
We confirm that PERMANOVA was performed as stated in our response. We have now integrated this analysis fully into the manuscript.

**In the Methods (Statistics, Section 2.6, Line 273-279，page 14):** We have added a new subsection to explicitly describe the PERMANOVA procedure:

"For multivariate analysis of β-diversity, Permutational Multivariate Analysis of Variance (PERMANOVA) was performed on the binary Jaccard distance matrix using the 'permanova' function with 9999 permutations. Pairwise PERMANOVA tests between groups were conducted where the overall model was significant, and p-values were adjusted for multiple comparisons using the Benjamini-Hochberg false discovery rate (FDR) correction."

**In the Results (Section 3.4.3, Line 376-384，page 19):** We have added the reporting of the PERMANOVA results:

"The PERMANOVA test, using 9999 permutations on a binary Jaccard distance matrix, indicated that grouping factor explained a significant portion of the variance in β-diversity (pseudo-F = 1.163, R² = 0.162, p = 0.001), with 15 samples distributed across 3 groups. Subsequent pairwise comparisons with FDR correction identified significant differences between NC group and HP group (p-adj = 0.003), and between NC group and HY001 group (p-adj = 0.018), while the difference between HP group and HY001 group was not significant (p-adj = 0.012). "

**In the Figure Legend (for the PCoA plot, Figure6):** We have added a note: "Statistical significance of group separation was assessed by PERMANOVA (pseudo-F = 1.163, R² = 0.162, p = 0.001)**."**

We believe these revisions fully address your concerns by providing a consistent methodological description and transparent statistical reporting. Thank you again for your meticulous review, which has significantly improved the clarity and rigor of our manuscript.

⸻

Minor Comments

**1. Taxonomic spelling and nomenclature**

Despite substantial improvement, several taxonomic spelling errors remain and should be corrected.

• In the Discussion: “Proteobactria” → “Proteobacteria”

• In the Abstract and Results: “unclassified-Muribaculaceaeae” → “unclassified-Muribaculaceae”

• In the Results: “Oscillospiracea” (Line 406) → “Oscillospiraceae”

**Response:** We sincerely thank the reviewer for their careful reading and for identifying these remaining taxonomic spelling errors. We have corrected each instance as specified, and the changes have been incorporated into the revised manuscript. The corrections are as follows:

**In the Discussion:**"Proteobactria" has been changed to "Proteobacteria".

**In the Abstract and Results:** "unclassified- Muribaculaceaeae" has been changed to "unclassified-Muribaculaceae".

**In the Results (Line 419):**"Oscillospiracea" has been changed to "Oscillospiraceae".

We have also performed a thorough, additional check of the entire manuscript to ensure consistency and accuracy in taxonomic nomenclature. Thank you again for helping us improve the precision of our manuscript.

**2. Other wording and typographical issues**

A few additional typographical and wording errors should be corrected for clarity and precision.

• Introduction (around line 83): “alleviating tail inflammation” should be corrected to “alleviating gastric inflammation”

• Methods (around lines 232–233): “Interleuki-8, Interleuki-1β, Interleuki-6” → “Interleukin-8, Interleukin-1β, Interleukin-6”

• Results (around line 371): “binary jaccard algorith”→ “binary Jaccard algorithm”

• Results (around line 364, IL-6 description)

The text currently states that the mean IL-6 concentration in the HY001 group is “higher than that of the HP group”, which contradicts the protective direction of effect described elsewhere and in the Discussion.

This is almost certainly a typo and should be corrected to “lower than that of the HP group”,

provided that the underlying data support this (if not, the interpretation in the Discussion should be adjusted accordingly).

• Figure 4 legend

The definitions of the HP and HY001 groups are currently confusing and partly duplicated, e.g.:

• “HP: treated by H. pylori SS1 group vs. pretreated by L. casei HY001 and treated by H. pylori SS1.”

• “HY001: treatment using L. case HY001 and treatment using H. pylori SS1; HP: treatment using H. pylori SS1.”

Please simplify and unify the legend.

• Figure 6 legend (gastric vs fecal)

In one place, the legend refers to the “composition of gastric microorganisms”, whereas in another location (around line 869) it refers to “composition of fecal microorganisms”. Since this study is based on gastric samples, all such references in the figure legends and text should be unified as “gastric microbiota” or “gastric microorganisms”, and any inadvertent “fecal” wording should be removed.

**Response:** Thank you for your thorough final review and for identifying these important typographical and wording errors. We have corrected each of them as specified, which has improved the clarity and precision of the manuscript. The changes are as follows:

**Introduction (around line 83-84)**: “alleviating tail inflammation” should be corrected to “alleviating gastric inflammation”.

**Methods (around lines 232–233)**: “Interleuki-8, Interleuki-1β, Interleuki-6” → “Interleukin-8, Interleukin-1β, Interleukin-6”.

**Results (around line 377)**: “binary jaccard algorith”→ “binary Jaccard algorithm”.

**Results (around line 370, IL-6 description).** The sentence in the Results section (around line 364) now accurately reflects our findings:

" Although the level of IL-6 was not statistically different in the HY001 group, its average concentration was already lower than that of the HP group."

**In the Figure 4 legend (Line 880-882, page 39),** The revised legend now reads as follows: **"** Group definitions: NC: Normal control group. HP: *H. pylori* SS1 infection group. HY001: *Lactobacillus casei* HY001 intervention group. HP+HY001: *H. pylori* SS1 infection followed by *L. casei* HY001 intervention group.**"**

**In the Figure 6 legend (Line 890-896, page 40),** the phrase "composition of fecal microorganisms" has been changed to **"composition of gastric microorganisms"**.

We have also performed a thorough check of the entire manuscript text and all figure legends to ensure that all references are consistently and accurately described as pertaining to **"gastric microbiota," "****gastric microorganisms, "**or**" gastric microbial communities."** Any other inadvertent uses of "fecal" in this context have been removed.

We apologize for this error and appreciate your careful attention to detail, which has helped us ensure the accuracy and consistency of our terminology.
